# Generalized global solar radiation forecasting model via cyber-secure deep federated learning

**DOI:** 10.1007/s11356-023-30224-1

**Published:** 2023-10-14

**Authors:** Arash Moradzadeh, Hamed Moayyed, Behnam Mohammadi-Ivatloo, António Pedro Aguiar, Amjad Anvari-Moghaddam, Zulkurnain Abdul-Malek

**Affiliations:** 1 Faculty of Electrical and Computer Engineering, University of Tabriz, Tabriz, 5166616471 Iran; 2 GECAD - Research Group on Intelligent Engineering and Computing for Advanced Innovation and Development, LASI - Intelligent Systems Associate Laboratory, Polytechnic of Porto, P-4200-072 Porto, Portugal; 3 School of Energy Systems, LUT University, Lappeenranta, Finland; 4 SYSTEC-ARISE Research Center for Systems and Technologies, Electrical and Computer Enginnering Department, Faculty of Engineering, University of Porto, P-4200 465 Porto, Portugal; 5 Department of Energy (AAU Energy), Aalborg University, 9220 Aalborg, Denmark; 6 Institute of High Voltage & High Current, Faculty of Electrical Engineering, Universiti Teknologi Malaysia, Johor Bahru, Malaysia

**Keywords:** Renewable energy resources, Solar energy, Solar radiation forecasting, Cyber-security, Federated learning, Deep learning, Machine learning

## Abstract

Recently, the increasing prevalence of solar energy in power and energy systems around the world has dramatically increased the importance of accurately predicting solar irradiance. However, the lack of access to data in many regions and the privacy concerns that can arise when collecting and transmitting data from distributed points to a central server pose challenges to current predictive techniques. This study proposes a global solar radiation forecasting approach based on federated learning (FL) and convolutional neural network (CNN). In addition to maintaining input data privacy, the proposed procedure can also be used as a global supermodel. In this paper, data related to eight regions of Iran with different climatic features are considered as CNN input for network training in each client. To test the effectiveness of the global supermodel, data related to three new regions of Iran named Abadeh, Jarqavieh, and Arak are used. It can be seen that the global forecasting supermodel was able to forecast solar radiation for Abadeh, Jarqavieh, and Arak regions with 95%, 92%, and 90% accuracy coefficients, respectively. Finally, in a comparative scenario, various conventional machine learning and deep learning models are employed to forecast solar radiation in each of the study regions. The results of the above approaches are compared and evaluated with the results of the proposed FL-based method. The results show that, since no training data were available from regions of Abadeh, Jarqavieh, and Arak, the conventional methods were not able to forecast solar radiation in these regions. This evaluation confirms the high ability of the presented FL approach to make acceptable predictions while preserving privacy and eliminating model reliance on training data.

## Introduction

Increasing energy demand and environmental issues have led to significant penetration of renewable energy sources (RESs) in the power systems and promoting the transition of energy structure (Dadashi et al. 2022). Solar energy has been introduced as a clean and permanent energy among RESs, so photovoltaic (PV) energy production is becoming an important part of the energy portfolio worldwide (Nasirpour et al. 2021; Rezazadeh et al. 2022). According to a statistical report through the International Energy Agency, 2.5% of the world’s total primary energy and 18.5% of RESs in 2019 have been supplied by solar energy (International Energy Agency (IEA) 2019). As indicated in another section of these statistics, the average annual growth rate of solar energy is 37%, which has created new possibilities and challenges for integration into existing power and energy systems (International Renewable Energy Agency 2020). However, the dependence of solar radiation on the synoptic meteorological pattern has caused the power generated by solar energy under PV energy technology to always have high variability and fluctuation (Wang et al. 2019a; Riahi et al. 2021). In addition, it causes some problems and inconsistencies related to the stabilization of energy resources and demand in electric power systems, particularly when the storage capacity of PV systems is low. Accordingly, solar radiation is a critical and effective parameter for solar energy applications. Awareness of the amount of solar radiation in different time periods can play an important role in the scheduling and planning of power network operators, residential solar energy operators, and industrial projects operators to manage their energy production and consumption (Bosman and Darling 2018; Sharda et al. 2021). Thus, the ability to forecast solar radiation is extremely important in terms of reliability and performance and is considered a critical factor in the penetration of solar energy into the power and energy networks. Also, accurate forecasts support the safe operation of the grid and allow maximum access to solar energy. To date, solar radiation forecasting based on various methods such as physical model (Caldas and Alonso-Suárez 2019; Kakimoto et al. 2019; Marzouq et al. 2020), statistical approach (Scolari et al. 2018; Van Der Meer et al. 2018; Louzazni et al. 2020), data mining-based solutions such as artificial neural network (ANN) (Khan et al. 2020; Wang et al. 2020a, b; Zambrano and Giraldo 2020), machine learning (Prasad et al. 2019; Deo et al. 2019; Yagli et al. 2019; Feng et al. 2019; Belmahdi et al. 2022), and deep learning (Wang et al. 2019b; Khodayar et al. 2020; Wen et al. 2021; Abdel-Nasser et al. 2021; Liu et al. 2022) techniques has been investigated in various studies.

Physical models forecast the solar radiation based on the principles of PV cell production and numerical weather prediction (NWP). In Caldas and Alonso-Suárez (2019), a novel model for short-term solar radiation forecasting via sky measurements and online imaging has been presented. In Marzouq et al. (2020), a multi-model evolutionary framework based on a physical model for solar radiation forecasting has been developed. A probabilistic approach to solar radiation prediction based on the joint probability distribution function (PDF) of the radiation calculated by NWP and the observed radiation was proposed in (Kakimoto et al. (2019). In the same study, the probabilistic forecast has been achieved by deriving a conditional PDF given a current NWP via the Bayes rule. The use of these methods is not cost-effective and requires additional peripherals such as sky cameras to collect input data. In addition, the collection of input data that determines the amount of solar radiation requires experienced people.

Statistical methodologies are usually suitable for time-series estimating of solar radiation. In Louzazni et al. (2020), the autoregressive moving average method is considered a statistical technique for forecasting solar radiation and PV output. In another similar work (Van Der Meer et al. 2018), an autoregressive integrated moving average model has been employed to forecast PV power production based on solar radiation. Two different mathematical methods and the Kalman filter technique are also proposed in (Scolari et al. (2018) as predictors of solar radiation. Despite their low cost, these methods have high mathematical computational complexity, and in most cases, the results of forecasts are not very accurate.

Data mining-based solutions make the necessary forecasts based on data affecting the amount of solar radiation. Each of these techniques has strengths and weaknesses in dealing with different datasets. One of the ANN techniques called direct explainable neural network, a forward-forward neural network, has been employed in Wang et al. (2020a) to forecast solar radiation at various time-horizons in Lyon, France. In Wang et al. (2020b), various ANN-based techniques for solar radiation prediction have been reviewed. In Zambrano and Giraldo (2020), an ANN-based procedure has been trained to forecast solar radiation with a horizon altering from 1 to 48 h utilizing full on-site data. A hybrid model based on multi-stage multivariate empirical mode disintegration combined with a random forest and ant colony optimization was created in Prasad et al. (2019) for solar radiation prediction. The developed algorithm performs the forecasting process in three steps including feature extraction, determining the best features, and finally forecasting the monthly solar radiation. Belmahdi et al. (2022) provide a complete assessment of the machine learning and time series methods used for solar radiation forecasting. In Yagli et al. (2019), the hourly solar radiation forecasting for 7 locations and 5 climatic zones in the continental United States has been done using 68 machine learning algorithms in a comparative approach. In a valuable study (Deo et al. 2019), another machine learning application called extreme learning machine has been selected to forecast long-term solar radiation in Australia. An unsupervised clustering-based solar forecasting procedure has been developed in Feng et al. (2019) for short-term global horizontal solar radiation forecasting. The suggested method in this study consists of three parts: pattern recognition, global horizontal radiation time series unsupervised clustering, and unsupervised clustering-based forecasting. The ANN-based and machine learning methods have been able to significantly address the shortcomings of prior techniques. However, these solutions suffer from the processing of high-dimensional and time-series data and do not have the ability to model the behavioral pattern of this data, which reduces the accuracy of forecasts.

In Abdel-Nasser et al. (2021), solar radiation forecasting was performed with a deep-learning application called long short-term memory (LSTM) and an aggregation function based on the Choquet integral employing six realistic datasets gathered at different locations in Finland. A multi-step solar forecasting scheme based on a convolutional neural network (CNN) has been introduced in Wen et al. (2021) for PV power ramp-rate control. In that study, in addition to meteorological data, stacked sky images that combine temporal-spatial information of cloud motions were utilized to improve forecasting performance. In Khodayar et al. (2020), a hybrid model called convolutional graph autoencoder, which combines two deep learning techniques, namely, the use of a CNN and autoencoders, has been presented to forecast solar radiation in multiple sites in a wide area in the northern states of the USA. An integrated precise short-term solar radiation forecasting model has been suggested in Wang et al. (2019b) based on the least absolute shrinkage and selection operator and LSTM. A novel solar radiation forecasting model based on the LSTM, variational Bayesian inference, and federated learning (FL) has been introduced in Zhang et al. (2020). In recent years, deep learning techniques with influence on power and energy system applications could solve problems related to machine learning methods in this field. However, these techniques often have difficulty processing real-time data that involve noise and require pre-processing. In addition, all reviewed forecasting methods have the ability to provide a local forecasting model and require training data from that area to provide forecasting in a region. To date, none of the studies has presented a global model with the ability to maintain data security and generalizability that can forecast solar radiation without the need for training data from a region.

**Fig. 1:**
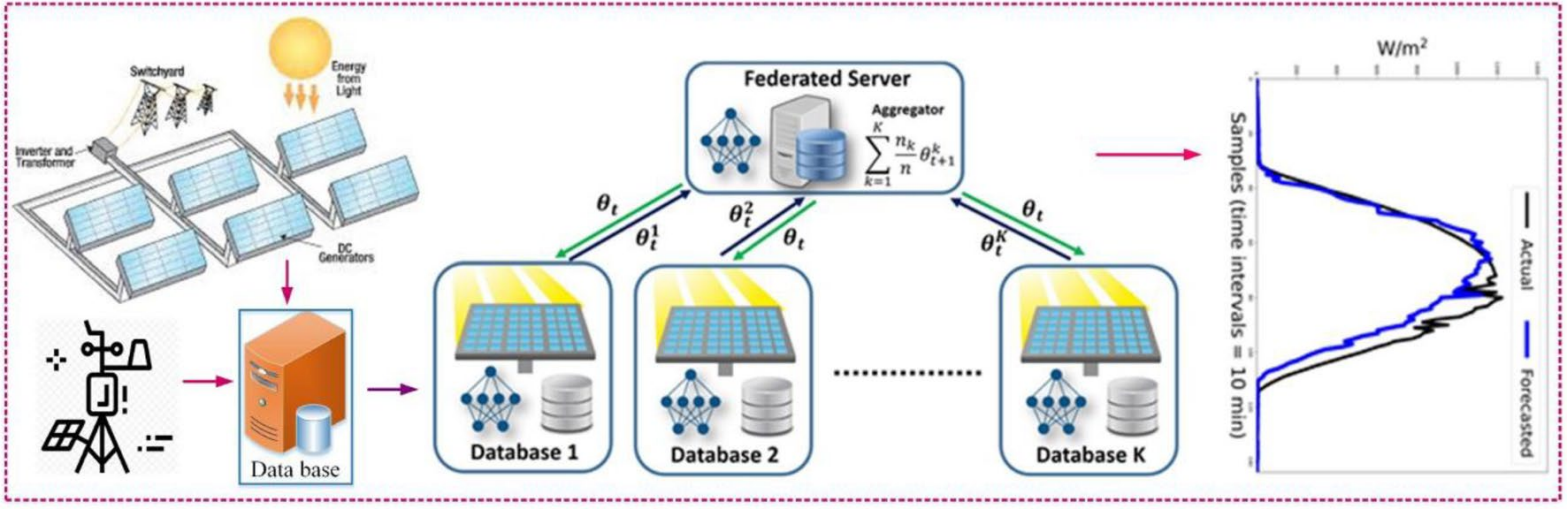
The overview of the proposed approach for solar radiation forecasting

In this study, a global solar radiation forecasting model with preserving data privacy is presented for the first time. The developed forecasting model is based on the FL approach, which uses the CNN technique in each client to accomplish the training cycle and extract features from the training data to form the main server, which is considered a global model. Each CNN network designed on each client of FL structure, while preserving the data security, extracting data-related features, and recognizing the pattern of solar radiation behavior at various time intervals. Then, based on the features extracted in each client, a global supermodel is generated. The proposed method is trained and tested using meteorological data related to different regions of Iran such as Birjand, Seydabad, Abhar, Mahan, Eqlid, Khaf, Meybod, and Tabriz that have solar power plants to produce the main server or global supermodel. To express the effectiveness of the proposed method and the performance of the global supermodel generated in solar radiation forecasting for regions whose data had no role in the formation of the server or global supermodel, meteorological data of new regions such as Abadeh, Jarqavieh, and Arak are used to test the global supermodel. In addition, in order to express the performance of the developed novel procedure against traditional models, conventional forecasting models such as decision tree (DT), back-propagation neural network (BPNN), LSTM, and bidirectional LSTM (Bi-LSTM) were also utilized to estimate solar radiation in the proposed regions. Figure 1 illustrates the overview of the solar radiation forecasting in this work.

In general, the most important contributions and novelty of this study are listed as follows:

(i) For the first time, an FL-based solar radiation forecasting model is suggested to offer a secure procedure for protecting data privacy by training the forecasting models locally and avoiding the exchange of raw data across various solar power plants.
(ii) The suggested procedure enables forecasting to benefit from the improved performance provided by global supermodel aggregation in the absence of data exchange.
(iii) Provide a generalizable model that is capable of forecasting solar radiation in various regions with different climatic conditions for which no training data are available. The higher generalizability of the proposed model is also substantiated while the privacy of the underlying data is preserved.
(iv) Various artificial intelligence-based techniques such as DT, BPNN, LSTM, and Bi-LSTM were used to forecast solar radiation in the study regions, in order to appraise the performance of the developed model compared to conventional models in a comparative approach.
(v) The evaluation of the results obtained is done for the proposed technique based on various performance evaluation indicators that indicate the high accuracy of the proposed FL-based solar radiation forecasting using real-world data sets.

The remainder of this paper is structured as follows: the methodologies used such as FL and CNN are described in the “Methodologies” section. The “Case studies” section introduces the study regions under the title of case studies. The “Solar radiation forecasting results” section presents the results of solar radiation forecasting. Finally, the paper is concluded in the “Conclusion” section.

## Methodologies

In this paper, the FL technique is proposed as one of the novel machine learning techniques to generate a global supermodel of solar radiation forecasting. The process of training and extracting features in each client from the federated network is done by one of the most well-known deep learning techniques called CNN. Conventional models of DT, BPNN, LSTM, and Bi-LSTM also are employed for solar radiation forecasting in the studied regions to provide a comparative approach for evaluating the results. The rest of this section introduces each of the developed forecasting procedures.

### Federated learning (FL)

Machine learning (ML) techniques including deep learning (DL) models are becoming increasingly complex as datasets grow, and there is a strong need to distribute the analysis process and optimization of model parameters across different machines. Moreover, current ML and DL models are trained for a specific dataset and in most cases they fail when analyzing observations that are not part of the data sample and have a different distribution. The generalization property is a desired feature for ML and DL models. Considering these properties, Google recently introduced the FL technique (Konečný et al. 2016), which proposes a communicated global model to link local ML models without seeing their location and, more significantly, without compromising privacy (Lu et al. 2021; Wang et al. 2021).

The main goal of the FL technique is to perform a global model with a partnership of several local parties (clients) that maintains the protection of the data while each local party is typically large and has Internet networks. This technique is called a decentralized privacy technique that allows clients to train their ML model without losing the privacy of the data. Furthermore, they do not need a central server to upload their local data. According to each iteration of the training, a distributed model is taken from the global server. Later, it is trained based on the local data, and eventually, the server gets the updated weights or gradients. The uploaded models by the clients must be aggregated to obtain a new overall model on the server.

FL has more robust local computational capabilities and more communication resources compared to traditional centralized learning. The local edges are taken into account when distributing the learning data, but the global server cloud does not have access to them. Nevertheless, the learned model is distributed to the clients and the server. Moreover, the local device is considered as the location for model training rather than the server. The server’s task is to aggregate the local models uploaded by the clients. The goal is to obtain a distributed global model and send it back to the clients (Lu et al. 2021; Moayyed et al. 2022).

**Fig. 2:**
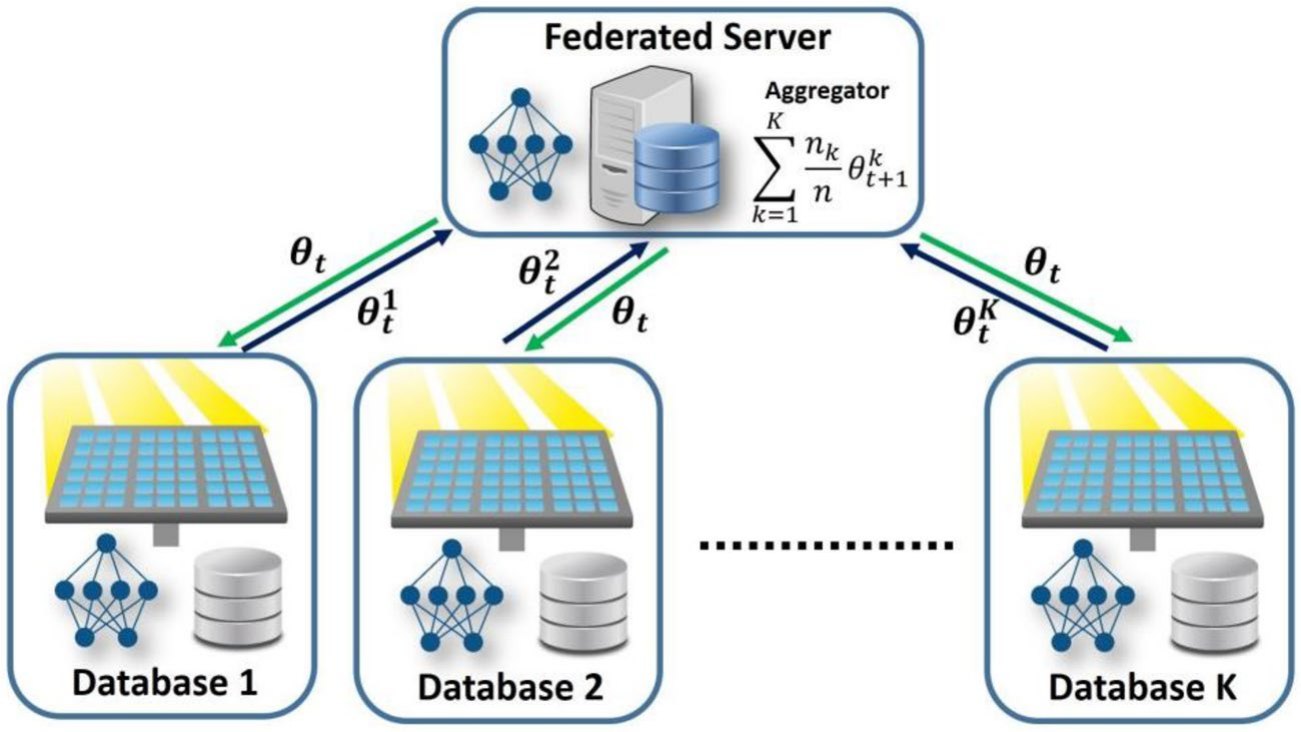
Architecture of FL model

**Fig. 3:**
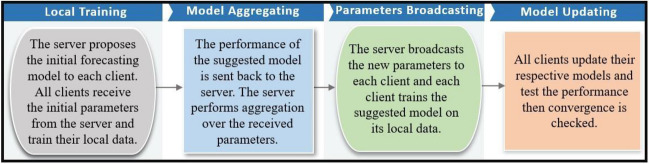
Implementation flowchart of the solar radiation forecasting process based on the developed FL method

A typical FL process is made up of one server and *K* clients, as shown in Fig. 2, and Fig. 3 shows the general process of implementing the developed FL method in the form of a flowchart. The FL algorithm is similar to the training algorithm of the multilayer perceptron neural network and intends to minimize the loss function *l*(*θ*) while working in a distributed scheme (Moayyed et al. 2022):

1 $$l\left(\theta \right)=\sum\limits_{k=1}^K\frac{n_k}{n}{L}_k\left(\theta \right)$$

where *K* denotes the number of total clients’ index and *L*_*k*_(*θ*) represents the loss function of the *k*th local client and can be formulated as:

2 $${L}_k\left(\theta \right)=\frac{1}{n_k}\sum\limits_{i\in {P}_k}{l}_i\left(\theta \right)$$

where *P*_*k*_ is considered a set of data indexes with the length of *n*_*k*_, i.e., *n*_*k*_ = |*P*_*k*_|.

As shown in Fig. 3, the implementation process of the developed model is possible in 4 steps as follows (Moradzadeh et al. 2021a):

- Step 1 (local training): All clients receive the parameters *θ*_*t*_ of the global model from the central server for local training. Then, the clients’ exclusive local models are trained by employing their own data.
- Step 2 (model aggregating): When the local parameters are trained, the server receives these parameters, such as ${\theta}_t^1$, from the local devices to perform secure aggregation across the local parties. The federated averaging algorithm (FedAvg) is the most common method for server aggregation (Kapur et al. 2011).
- Step 3 (parameter distribution): An updated global model *θ*_*t* + 1_ is broadcasted for the next iteration’s training;
- Step 4 (model updating): All clients update their local ML models based on the parameters received from the server and analyze the efficacy of the current models.

### Convolutional neural network (CNN)

**Fig. 4:**
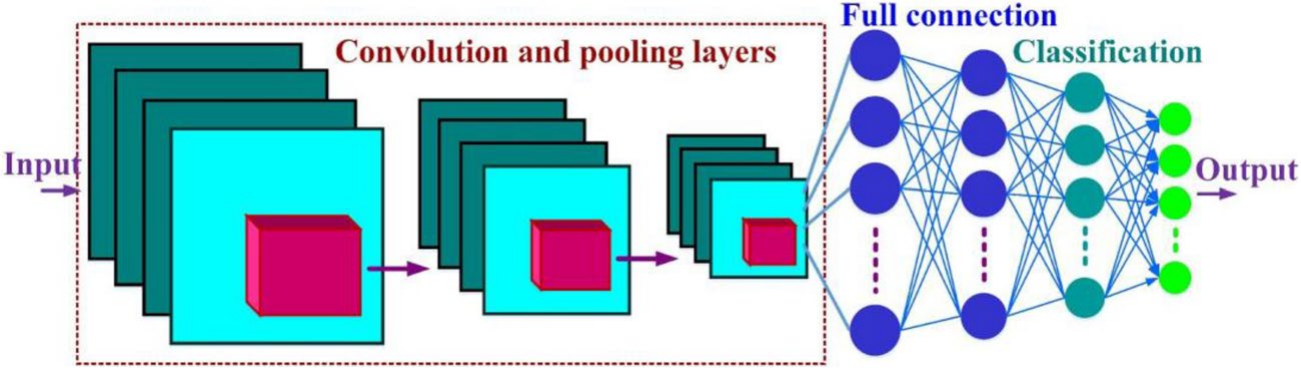
General architecture of CNN

The CNN is a subset of powerful deep learning techniques that have been used in recent years mainly in scientific and industrial applications associated with energy and power systems. Feature extraction, image processing, pattern recognition, categorization, and forecasting are some of the obvious capabilities of this scheme (Moradzadeh et al. 2021d). As Fig. 4 shows, the CNN structure consists of convolution layers, pooling layers, fully connected layers, and finally classification layers. The deep and layer-by-layer structure of CNN makes it possible to extract local features in the lower layers and then combine them into more abstract features in the higher layers (Moradzadeh et al. 2021b).

The convolution layer consists of a trainable bias and a set of learnable kernels that act as a filter to find the main characteristics from the input data. Each kernel, in proportion to its size, extracts the features from the data to eventually form a feature map in each convolution layer. The output of neurons that are connected to the input volume is obtained by calculating the product of the point between their weight and the small area. In each convolution layer, a nonlinear activation function called rectified linear unit (ReLU) is employed as (0, *x*) (Moradzadeh et al. 2021d). The *l*-layer convolved feature maps in a CNN structure with *L*-layers can be calculated as follows (Moradzadeh et al. 2021b):

3 $${y}_{l,j}^{conv}=\sum\limits_i^k{w}_{i,j}^l\ {y}_{l-1,i}^{pool}+{b}_j^l$$

4 $${y}_{l,j}^{ReLU}=f\left({y}_{l,j}^{conv}\right)=\mathit{\max}\left[0,{y}_{l-1,j}^{conv}\right]$$

where ${y}_{l,j}^{conv}$ and ${w}_{i,j}^l$ are the output and convolutional kernel related to the *l*th layer, respectively. *k* shows the number of kernels and ${b}_j^l$ represents the bias. *f*(·) is the activation function.

After feature extraction by different kernels, a pooling layer is added to each convolution layer to perform the pooling operation to collect the features and generate the feature map. Pooling is a type of nonlinear down-sampling that can reduce each map size and achieve spatial invariance. Pooling strategy can be done in two ways, average and max pooling. The max pooling is mainly used due to advantages such as leading to faster convergence, selection of superior invariant features, and improved generalization. The max pooling operation is obtained as follows (Moradzadeh et al. 2021d):

5 $${y}_{l,j}^{pool}={\mathit{\max}}_{M\times N}\left(p\left({S}_1,{S}_2\right){y}_{l-1,j}^{ReLU}\right)$$

where *p*(*S*_1_, *S*_2_) shows the window function used for the stored data and *S*_1_ and *S*_2_ depict the window size, which can be of arbitrary size and overlapping. *M* × *N* corresponds to the size related to the *l*-layer feature map output.

After the last convolution layer in the CNN structure, there are fully connected layers that act like a feed-forward neural network. Output of the last convolution layer, the last feature map formed, is applied as the input of fully connected layers. At this stage, the extracted features, after determining the weight and bias, are transferred to the last layer to estimate the final output of the CNN network. The last layer is a Softmax layer that forecasts the final output of the network based on the specified targets (Moradzadeh et al. 2021b).

### Decision tree (DT)

**Fig. 5:**
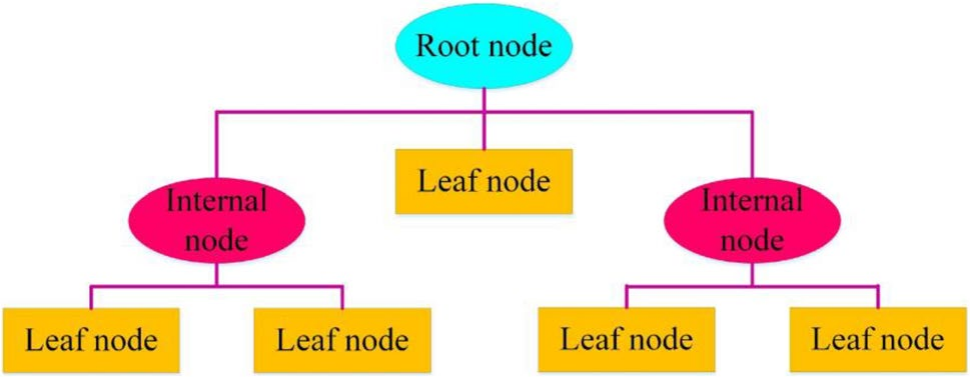
The architecture of DT

The decision tree is one of the most commonly applied techniques of machine learning, which is based on inductive learning and is introduced in the form of a systematic method called recursive binary partition. Linear regression, pattern recognition, classification, and forecasting are obvious applications of this method (Moradzadeh et al. 2021e; Latif 2021). Like the model shown in Fig. 5, tree-shaped diagrams form the composition of decision trees. This architecture consists of branches and three types of nodes named root node, inner node, and leaf node (Moradzadeh et al. 2021e). In the implementation of the training process, the input data is divided into small categories or a number of subsets with the help of some dichotomous classifications. Nodes, which are points in a tree, are responsible for processing features. Branches are also test results and are responsible for forming subsequent nodes in this architecture. The root node is the highest node, the inner nodes are in the middle of the DT structure, and the leaf nodes are considered the end nodes. Reaching a node to a predetermined category purity level and achieving only a single output per node completes the training and test process of the DT (Namazkhan et al. 2020).

### Back-propagation neural network (BPNN)

**Fig. 6:**
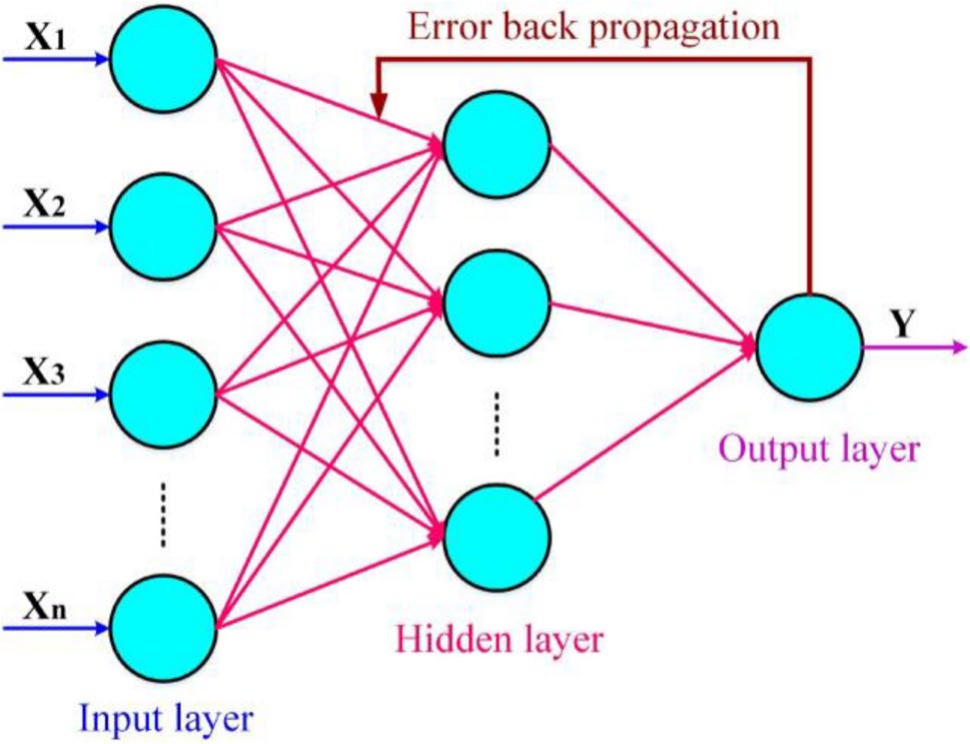
The architecture of BPNN

BPNN is one of the most well-known applications of ANNs, first introduced in 1972 by Parker. This algorithm is structurally and functionally slightly different from a feed-forward neural network (FFNN) and has significant applications in various sciences and industries (Yokoyama et al. 2009). Like the structure presented in Fig. 6, the BPNN consists of an input layer, a hidden layer, and an output layer in its structure which the existence of a back propagation has caused the structural difference between this network and FFNNs (Moradzadeh et al. 2022a).

In this structure, the letters *m*, *T*, and *n* represent the number of nodes in the input layer, hidden layer, and output layer, respectively. *w*_*ij*_ and *w*_*jk*_ denote the connection weight in the layers, and *X* and *Y* represent the input values and the forecasting values, respectively. The input data is received as a signal by the input layer and after processing is transferred to the hidden layer. The hidden layer, as an internal processing layer, has the task of processing and sending signals to the output layer. The training process of ANN-based algorithms requires an activation function, which in this paper, *f*(*x*) is used as a sigmoid activation function (Chaudhuri et al. 2019):

6 $$f(x)=\frac{1}{\left(1+{e}^{-x}\right)}$$

In the input layer, the output related to each neuron is computed as follows (Moradzadeh et al. 2022a):

7 $${O}_{ik}=f\left(\sum\limits_{j=0}^l{W}_{kj}{O}_{ij}\right),k=1,2,\dots, n$$

where the connection weight from the *j*th input neuron to *k*th hidden neuron is denoted by *W*_*kj*_. Finally, the network output for *n* hidden neurons is forecasted according to the following equation (Moradzadeh et al. 2022a):

8 $${Y}_i=f\left(\sum\limits_{k=0}^n{W}_k{O}_{ik}\right),k=1,2,\dots, n$$

### Long short-term memory (LSTM)

**Fig. 7:**
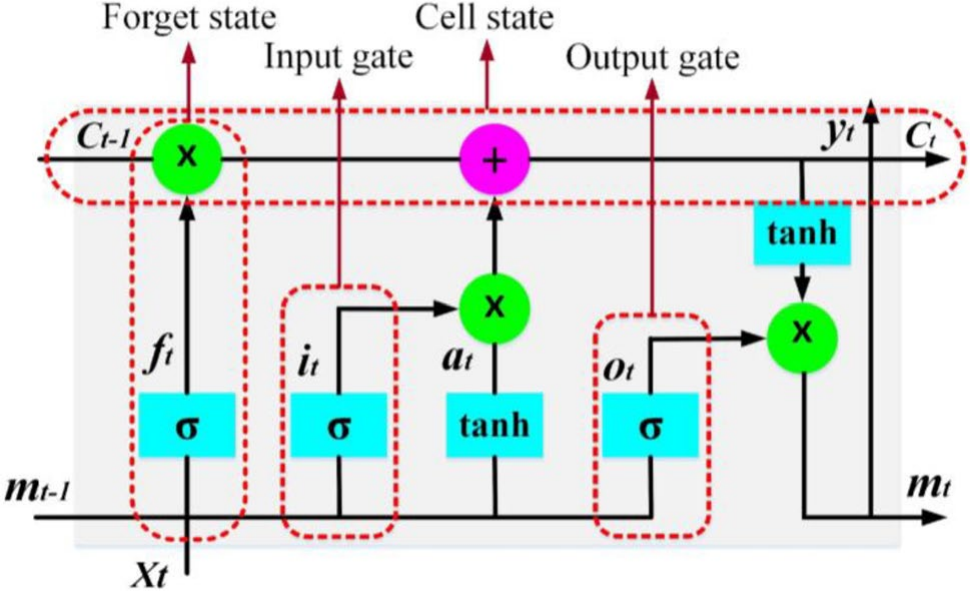
Structural schematic of an LSTM unit

LSTM network is one of the deep learning techniques that has been proposed to mitigate the vanishing/exploding gradient problems related to RNN networks. The LSTM uses a memory unit to store dependent information in consecutive data for a long horizon time (Moradzadeh et al. 2020; Fu et al. 2021). Nonlinear data analysis, regression, estimation of the relationship between input and output variables, and classification are the salient applications of LSTM. Figure 7 shows the structure of an LSTM unit, which consists of a forget gate, input gate and output gate, and a memory cell (Moradzadeh et al. 2022d).

The presence of an intrinsic memory function in this structure allows the network to preserve pre-extracted information. The cell state update in each LSTM unit is done based on the results of the forget gate and input gate. The training process of this network is mainly done using algorithms such as gradient descent and back-propagation. Mathematical modeling of LSTM architecture is as follows (Moradzadeh et al. 2022c):

9 $${f}_t=\sigma \left({W}_{lf}{l}_t+{W}_{mf}{m}_{t-1}+{b}_f\right)$$

10 $${i}_t=\sigma \left({W}_{li}{l}_t+{W}_{mi}{m}_{t-1}+{b}_i\right)$$

11 $${o}_t=\sigma \left({W}_{lo}{l}_t+{W}_{mo}{m}_{t-1}+{b}_o\right)$$

12 $${a}_t=\mathit{\tanh}\left({W}_{la}{l}_t+{W}_{ma}{m}_{t-1}+{b}_a\right)$$

13 $${c}_t={c}_{t-1}\upphi {f}_t+{i}_t\upphi {a}_t$$

14 $${m}_t={o}_t\upphi \mathit{\tanh}{c}_t$$

where *σ* is the activation function called logistic sigmoid. Each of the forget, input, and output gates are denoted by *f*_*t*_, *i*_*t*_, and *o*_*t*_, respectively. *c*_*t*_ shows the memory cell and *a*_*t*_ demonstrates the hidden vector. *W*_*l*∗_ = {*W*_*lf*_, *W*_*li*_, *W*_*la*_, *W*_*lo*_} and *W*_*m*∗_ = {*W*_*mf*_, *W*_*mi*_, *W*_*ma*_, *W*_*mo*_} represent the weights related to corresponding gates. *b*_*f*_, *b*_*i*_, *b*_*o*_, and *b*_*a*_ are output biases. ɸ is the Hadamard product operator.

### Bidirectional long short-term memory (Bi-LSTM)

**Fig. 8:**
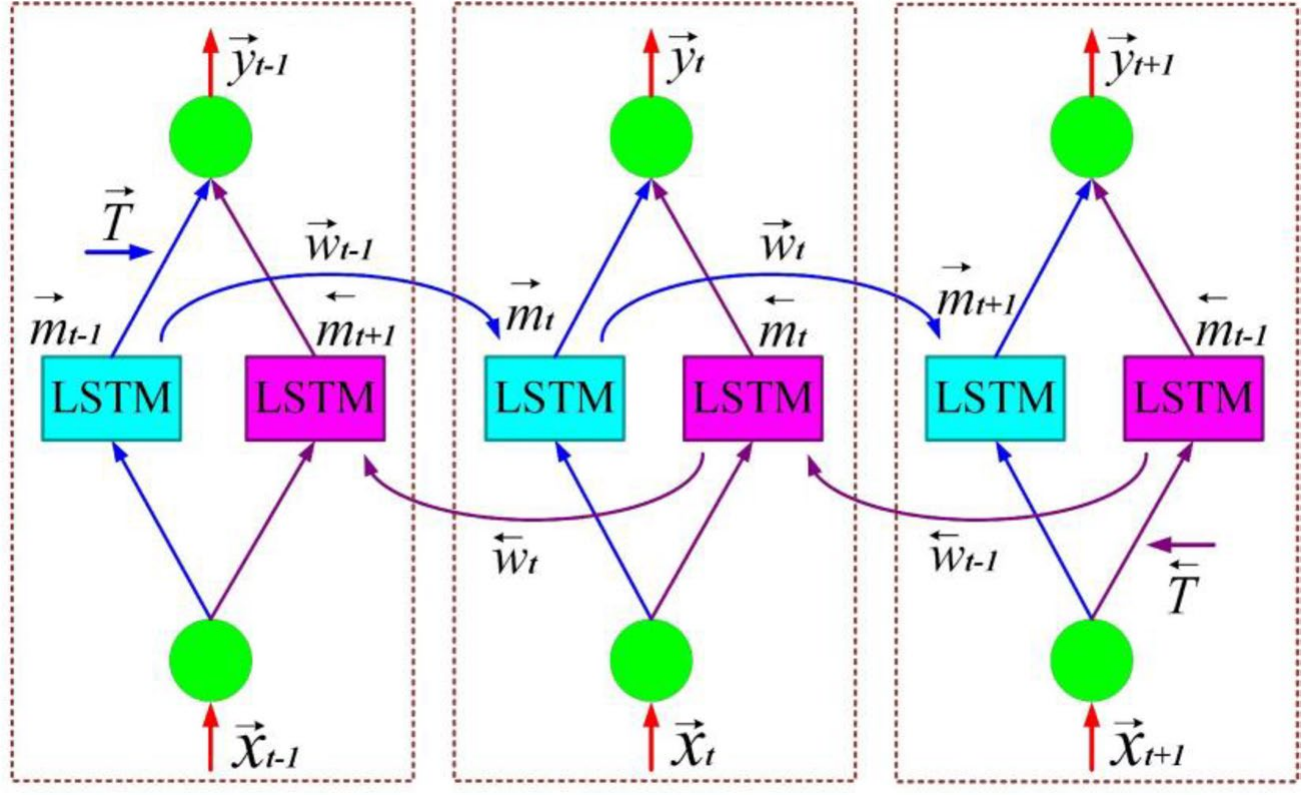
The structure of Bi-LSTM

As an advanced type of LSTM, the Bi-LSTM is developed to model time-series state of data and process large volumes of data. Like the architecture presented for the Bi-LSTM network in Fig. 8, the two-way training process, which includes forward and backward layers, distinguishes the Bi-LSTM architecture from LSTM (Moradzadeh et al. 2021c). Thus, the transfer of information during the training process of this network is done as a two-way process forward (blue line) and reverse (purple line) that can use the past and current information associated with the data to train the next data. The two-direction calculation of the hidden layer and the output layer of Bi-LSTM at time *t* is as follows (Das et al. 2020; Moradzadeh et al. 2022b):

15 $${\overrightarrow{m}}_t=\sigma \left({\overrightarrow{W}}_i{x}_t+{\overrightarrow{V}}_i{\overrightarrow{m}}_{t-1}+\overrightarrow{b}\right)$$

16 $${m^{\leftarrow}}_t=\sigma \left({W^{\leftarrow}}_i{x}_t+{V^{\leftarrow}}_i{m}_{t+1}+{b}^{\leftarrow}\right)$$

17 $${y}_t=\sigma \left(U\left[{\overrightarrow{m}}_t;{m}_t\right]+c\right)$$

Evaluating the performance of each of the Bi-LSTM and LSTM networks in various studies has emphasized the high capability of Bi-LSTM, citing some of its structural advantages, such as the law of learning forward and backward. Today, the development of power systems and the increasing use of measuring equipment and communication devices have resulted in the production of large volumes of data that include certain behavioral patterns of the power system over long periods of time. Hence, the Bi-LSTM network, due to its capabilities in processing large volumes of data and modeling the time-series state of data, can be significantly used in power system applications.

## Case studies

**Fig. 9:**
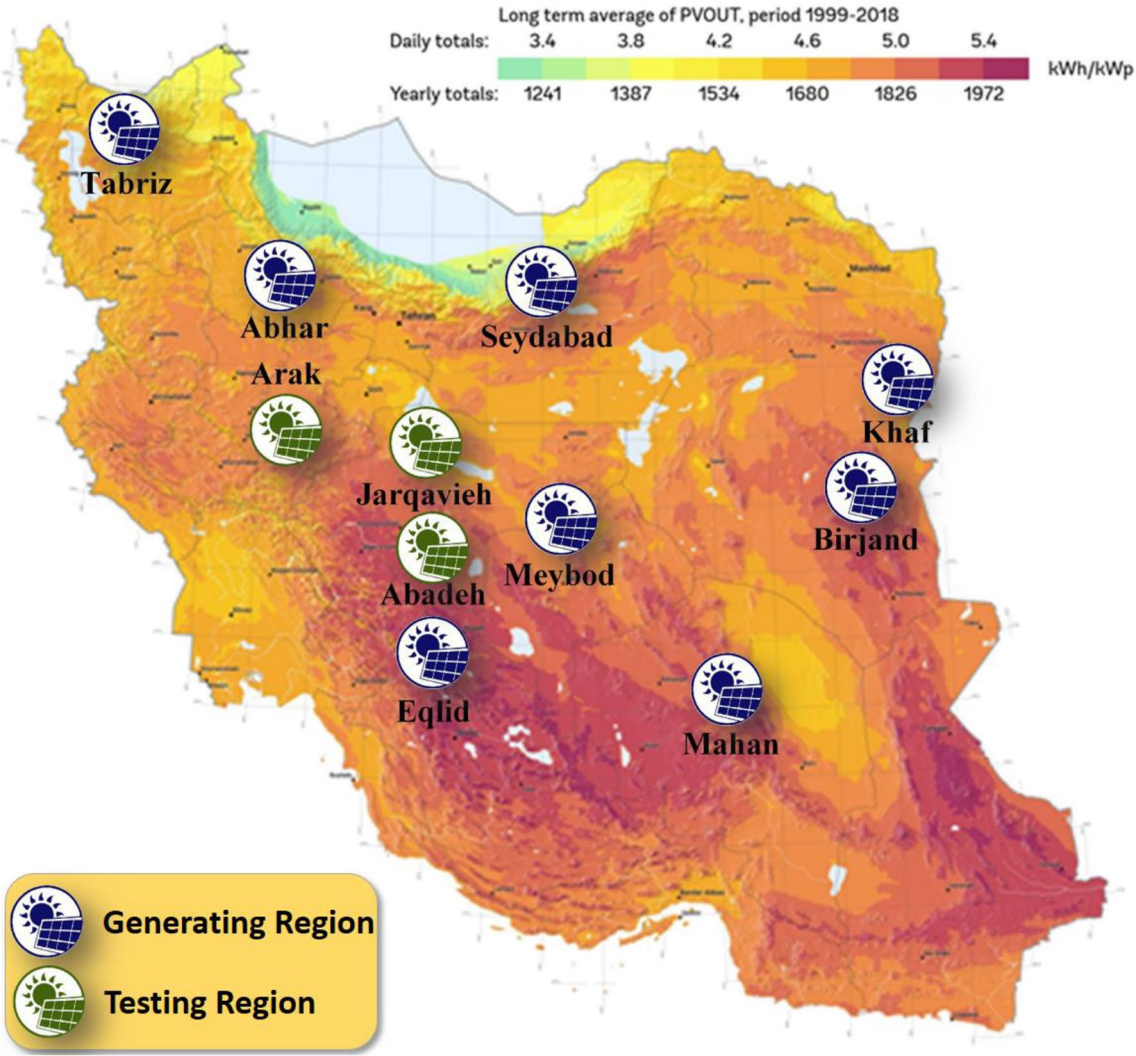
Locations of considered meteorological stations within Iran’s solar radiation map

In this paper, solar radiation forecasting is done for regions of Iran that have solar power plants. As shown in Fig. 9, the regions studied for training and generating supermodel, as well as new regions for testing the generated supermodel, are selected from various areas of Iran with various climatic and geographical features. In each client of the federated network, the CNN is trained using data from regions of Birjand, Seydabad, Abhar, Mahan, Eqlid, Khaf, Meybod, and Tabriz and extracts the pattern of solar radiation behavior for different regions. Then, based on the extracted pattern, it forms the global supermodel. Data for regions Abadeh, Jarqavieh, and Arak that did not exist in each client training dataset are utilized as anonymous data to test the global supermodel. In each region, data collected by meteorological stations consisting of humidity, temperature, wind speed and direction, and solar radiation with the sampling intervals of 10 min were used.

## Solar radiation forecasting results

The solar radiation forecasting in this study is done based on the FL and CNN schemes. It is a technique that, while preserving the privacy of the data, by training with dataset related to 8 scattered regions of Iran, provides a global supermodel that can respond to forecast the solar radiation for all regions of Iran. A CNN procedure has been employed to train the server on each client. To build the FL algorithm, the authors did not use any prepared library based on FL and constructed the model to drive the server, the aggregation process, and broadcasting models to the clients. The CNN was developed employing the TensorFlow deep learning framework and trained to operate the Adam optimization algorithm and the mean squared error loss function. The CNN network consists of 2 convolution layers with 64 and 128 filters and kernel size 3. Three dense layers complete the network. These layers are equipped with activation functions of ReLU. The data for Birjand, Seydabad, Abhar, Mahan, Eqlid, Khaf, Meybod, and Tabriz regions in each client is used to train and test the CNN as an input dataset. In each dataset, time variables, such as date and hour of the day, and climatic factors including wind speed and direction, humidity, and temperature with the sampling intervals of 10 min are considered input variables and the solar radiation parameters as output or target variables. Eighty percent of each dataset is considered for network training and recognition of solar radiation behavior patterns and 20% of the data is selected to test the CNN in each client. Following the completion of the network training and validation process, the federated network will generate a global supermodel based on the features extracted by the CNN. This supermodel, which is also the main objective of this study, is able to forecast the solar radiation for new regions from anywhere in Iran. Accordingly, to test the global supermodel and illustrate its effectiveness, data from new regions of Iran named Abadeh, Jarqavieh, and Arak are utilized to forecast solar radiation. Table 1 shows the number of samples in each region and the number of samples intended for training and testing the CNN in each client and testing the global supermodel generated.

**Table 1:** The total stored data was dedicated to training and testing from each region

	Location	No. of samples	No. of training	No. of tests	Start	End
1	Birjand	13,392	10,714	2678	22/06/2007	22/09/2007
2	Seydabad	13,392	10,714	2678	22/06/2007	22/09/2007
3	Abhar	13,324	10,660	2664	07/07/2015	07/10/2015
4	Mahan	13,392	10,714	2678	01/07/2015	01/10/2015
5	Eqlid	13,073	10,459	2614	24/06/2006	22/09/2006
6	Khaf	13,104	10,484	2620	09/07/2007	07/10/2007
7	Meybod	13,392	10,714	2678	22/06/2015	22/09/2015
8	Tabriz	4649	3719	930	22/06/2011	11/09/2011
9	Abadeh	5721	–	5721	22/06/2006	31/07/2006
10	Jarqavieh	5691	–	5691	24/06/2006	02/08/2006
11	Arak	8281	–	8281	07/05/2006	31/08/2006

At each stage of training and test, the CNN results are evaluated for each client based on a variety of performance evaluation indicators. These indicators include mean square error (MSE), root mean square error (RMSE), mean absolute error (MAE), mean absolute percentage error (MAPE), and correlation coefficient (*R*^2^) each of which expresses a specific concept of network performance and forecasts of solar radiation. These metrics were also employed to appraise the performance of the supermodel. Each of the above indicators is computed based on the following equations (Moradzadeh et al. 2022c):

18 $$MSE=\frac{1}{N}\sum\limits_{i=1}^N{\left({X}_i-{Y}_i\right)}^2$$

19 $$RMSE=\sqrt{\frac{1}{N}\sum\limits_{i=1}^N{\left({X}_i-{Y}_i\right)}^2}$$

20 $$MAE=\frac{1}{N}\sum\limits_{i=1}^N\left|\left({X}_i-{Y}_i\right)\right|$$

21 $$MAPE=\frac{1}{N}\sum\limits_{i=1}^N\left|\frac{X_i-{Y}_i}{X_i}\right|\times 100$$

22 $${R}^2=\frac{\sum\limits_{i=1}^N\left({X}_i-\underset{\_}{X}\right)\left({Y}_i-\underset{\_}{Y}\right)}{\sqrt{\sum\limits_{i=1}^N{\left({X}_i-\underset{\_}{X}\right)}^2\sum\limits_{i=1}^N{\left({Y}_i-\underset{\_}{Y}\right)}^2}}$$

where *X*_*i*_ and *Y*_*i*_ are the real solar radiation parameters and forecasted solar radiation parameters, respectively. $\underline X$ and $\underline {Y}$ denote the average of real solar radiation parameters and average of forecasted solar radiation parameters, respectively.

Each of the mentioned statistical indicators evaluates the performance of the desired network with a specific concept. In general, indicators MSE, RMSE, MAE, and MAPE are related to forecast error, and in these indicators, values close to zero are ideal. The *R*^2^ index indicates the accuracy of the network and it is better to have its values close to 1. Each CNN network is trained in each client based on data from the intended regions. At the end of the training process, each network is saved as a toolbox to be used to forecast the test data. As mentioned, at this stage, each trained CNN network is tested by 20% of the data from the 8 regions mentioned. Table 2 presents the results of solar radiation forecasting for the regions intended for supermodel generation in the test stage according to the mentioned evaluation indicators. The high accuracy and excellent performance of the CNN model in training and the test process of data related to each region can be seen from the results presented in Table 2.

**Table 2:** Performance of server for test data of 8 clients

Region	MSE	RMSE	MAE	MAPE	*R*^2^
Birjand	0.0016	0.040	0.018	1.33	0.98
Seydabad	0.0047	0.069	0.036	2.54	0.95
Abhar	0.0043	0.066	0.038	2.92	0.96
Mahan	0.0067	0.082	0.039	2.75	0.94
Eqlid	0.0047	0.069	0.041	3.33	0.96
Khaf	0.0022	0.047	0.020	1.30	0.98
Meybod	0.0019	0.044	0.020	1.36	0.98
Tabriz	0.0079	0.089	0.042	3.11	0.94

**Fig. 10:**
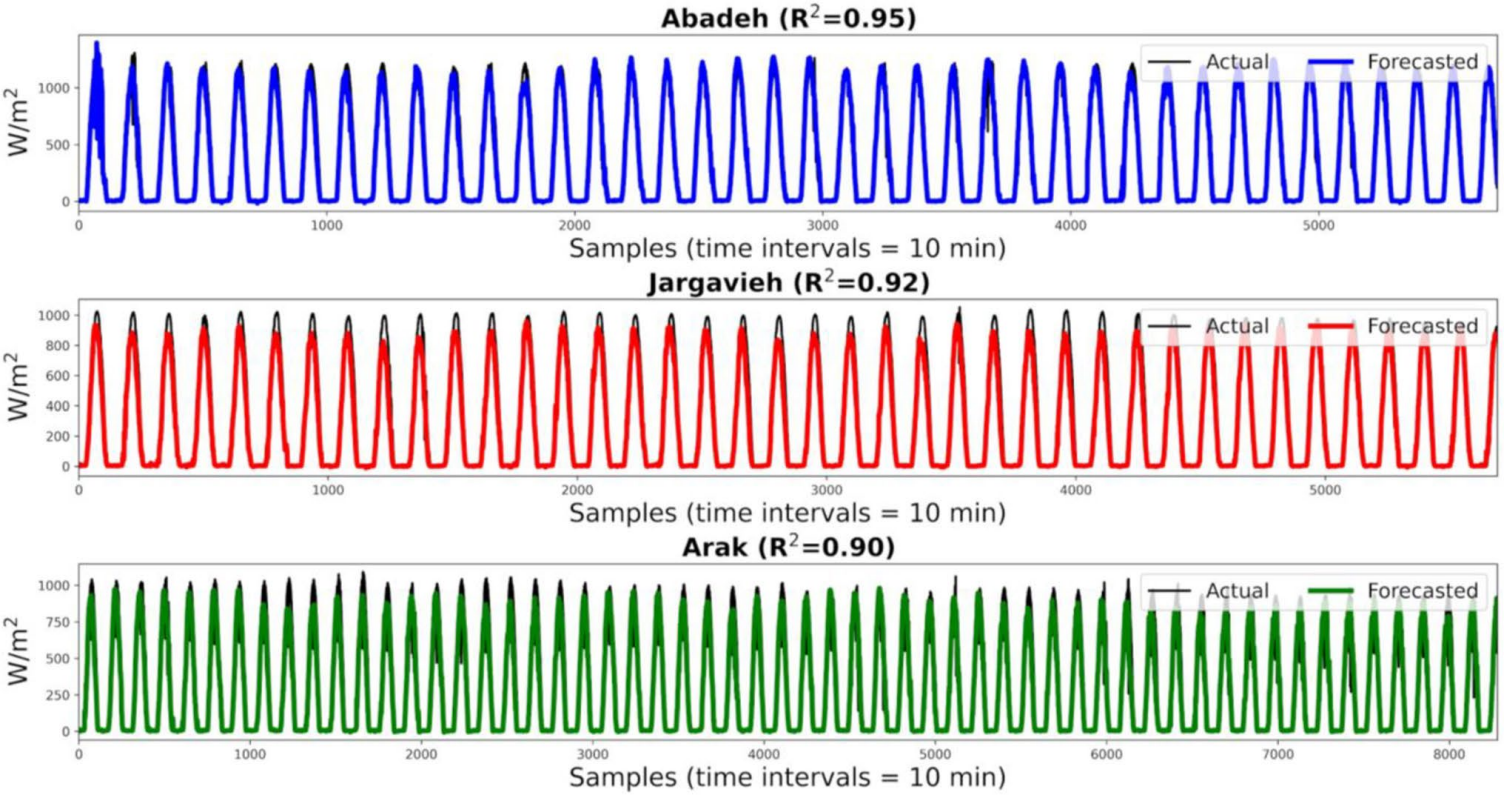
Comparison of actual and forecasted solar radiation values by the supermodel for the three new regions based on the *R*^2^ evaluation index

**Fig. 11:**
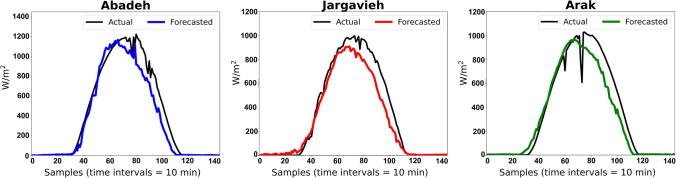
Examples of 1-day (24 h) solar radiation forecasts from each new region by the global supermodel

The results show that the CNN in each client has been able to train the behavioral pattern of solar radiation well and to forecast its values based on input variables from each region with high correlation. Based on this training and test process, the federated network generates a global supermodel that contains features extracted in each client. The supermodel produced should be able to forecast solar radiation for regions that have not been included in the training process. To do this, data from regions of Abadeh, Jarqavieh, and Arak are fully utilized (as shown in Table 1) to test the generated global supermodel. Figure 10 shows the real values of solar radiation and related results of forecasting solar radiation using the supermodel generated for the three new regions. In this figure, the correlation between the actual parameters and the forecasted parameters of solar radiation based on the *R*^2^ index is presented. In order to express the effectiveness of the global supermodel, predictive samples for 1 day (24 h) are randomly provided from each region with high resolution in Fig. 11. It can be seen that although no data from these three regions is used in the process of server training in any client, the supermodel is able to provide an acceptable forecast of solar radiation in these regions, namely, Abadeh, Jarqavieh, and Arak, with *R*^2^ values of 0.95, 0.92, and 0.90, respectively.

**Fig. 12:**
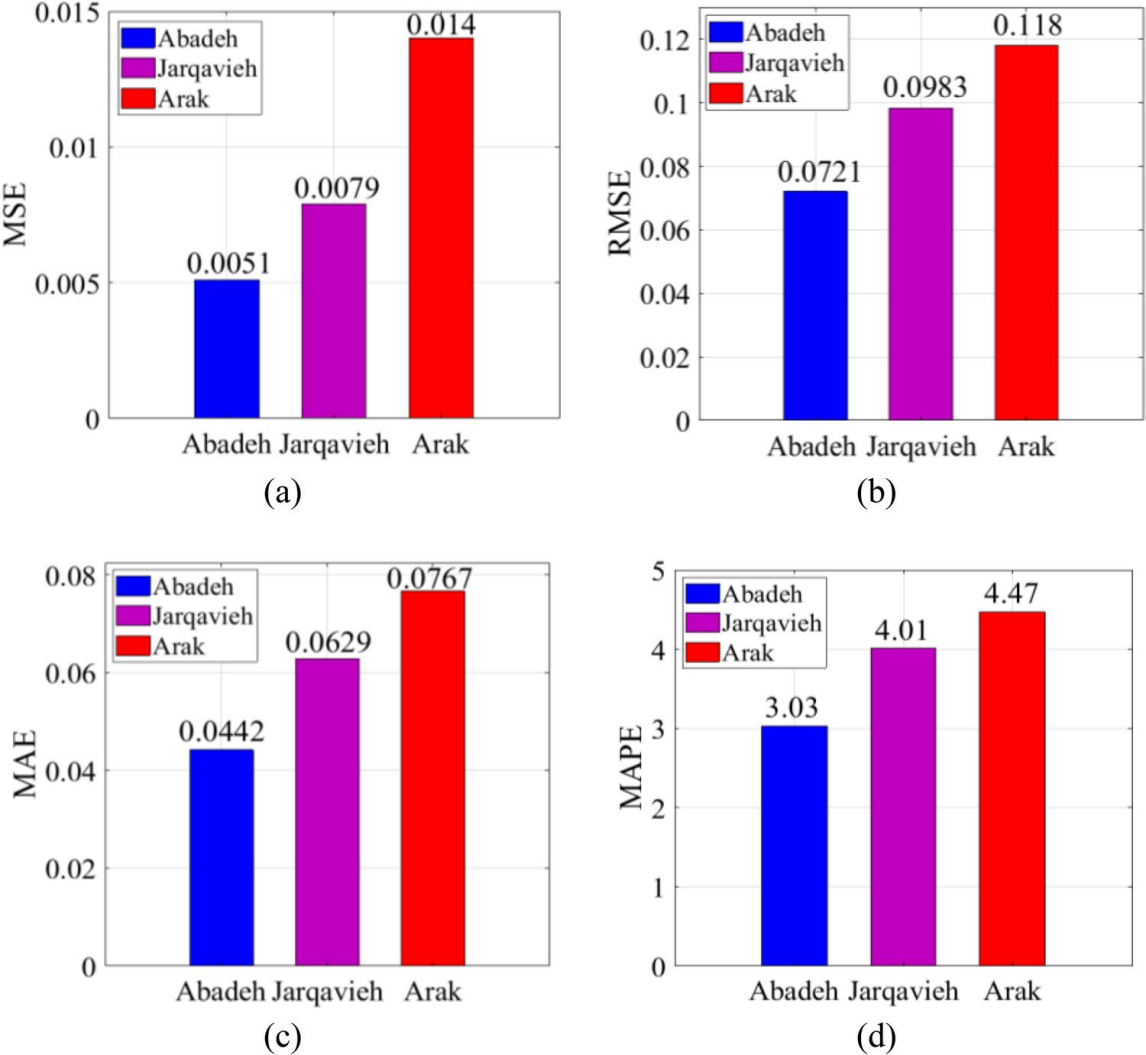
Performance evaluation of global supermodel in forecasting solar radiation for new regions: **a** MSE, **b** RMSE, **c** MAE, **d** MAPE

Fig. 12 evaluates the forecasting results presented by the global supermodel based on statistical indices MSE, RMSE, MAE, and MAPE, respectively. The evaluation of the results in Fig. 12 has been performed based on various statistical indicators and it can be seen that the supermodel is able to perform the prediction process for each of the new regions with low and acceptable error values. Among the new regions, the forecasts made for the Abadeh region, which also has a higher solar energy potential (as shown in Fig. 9) than two other regions, have the highest accuracy score and the lowest forecasting error values. The supermodel is also able to forecast the solar radiation for the region of Jarqavieh with a higher accuracy coefficient than the Arak. Figure 9 also shows that the region of Jarqavieh has a higher solar energy potential than the Arak region. The results show that the proposed approach can be used as a powerful tool for power grid and renewable energy operators where privacy is a high priority.

As stated in the literature, various conventional methods such as DT, BPNN, LSTM, and Bi-LSTM were also employed to forecast solar radiation in the study regions to make a comparative assessment of the performance of the developed FL method. Table 3 evaluates and compares the results of DT, BPNN, LSTM, and Bi-LSTM methods in solar radiation forecasting.

**Table 3:** Analyzing and comparing the results of methods DT, BPNN, LSTM, and Bi-LSTM in solar radiation forecasting

Region	DT					BPNN					LSTM					Bi-LSTM				
	MSE	RMSE	MAE	MAPE	*R*^2^	MSE	RMSE	MAE	MAPE	*R*^2^	MSE	RMSE	MAE	MAPE	*R*^2^	MSE	RMSE	MAE	MAPE	*R*^2^
Birjand	0.0027	0.051	0.024	1.47	0.97	0.0033	0.057	0.029	1.52	0.97	0.0019	0.043	0.020	1.39	0.97	0.0013	0.036	0.017	1.31	0.98
Seydabad	0.0051	0.071	0.039	2.60	0.94	0.0054	0.073	0.042	2.65	0.93	0.0046	0.067	0.034	2.52	0.95	0.0043	0.065	0.034	2.51	0.96
Abhar	0.0054	0.073	0.043	2.92	0.94	0.0051	0.071	0.039	2.58	0.94	0.0046	0.067	0.042	2.97	0.95	0.0044	0.066	0.040	2.96	0.96
Mahan	0.0070	0.083	0.044	2.93	0.93	0.0072	0.084	0.045	2.90	0.94	0.0075	0.086	0.047	2.84	0.93	0.0060	0.077	0.031	2.72	0.95
Eqlid	0.0063	0.079	0.058	3.40	0.93	0.0065	0.080	0.060	3.39	0.93	0.0058	0.076	0.053	3.49	0.94	0.0046	0.067	0.040	3.29	0.96
Khaf	0.0037	0.060	0.034	1.54	0.97	0.0044	0.066	0.039	1.61	0.96	0.0031	0.055	0.029	1.47	0.97	0.0020	0.044	0.019	1.24	0.98
Meybod	0.0040	0.063	0.039	1.61	0.97	0.0042	0.064	0.043	1.67	0.97	0.0027	0.051	0.027	1.43	0.98	0.0024	0.048	0.023	1.41	0.97
Abadeh	Failed					Failed					Failed					Failed				
Jarqavieh	Failed					Failed					Failed					Failed				
Arak	Failed					Failed					Failed					Failed				

The presented results in Table 3 show the unique ability of each method to forecast solar radiation in different regions. It can be seen that the Bi-LSTM method was superior to DT, BPNN, and LSTM models and had the least forecasting error values compared to other methods. However, the lack of generalizability of conventional methods has made none of the DT, BPNN, LSTM, and Bi-LSTM models able to provide forecasts in regions Abadeh, Jarqavieh, and Arak. Meanwhile, the developed FL method has been able to forecast the solar radiation in regions Abadeh, Jarqavieh, and Arak, with the accuracy of 95%, 92%, and 90%, respectively, under the same data distribution conditions as conventional methods. Accordingly, due to the fact that meteorological stations are not available in all parts of the country, the proposed method can be used effectively for industrial projects that are in the direction of operation of RESs, especially in remote areas. In addition, the FL scheme can be employed for all forecasting problems in power and energy systems.

## Conclusion

Optimal operation and management of renewable energy sources (RESs), especially solar energy, need a comprehensive database to construct precise models for forecasting the behavior of solar radiation in diverse regions and time spans. Because environmental data and solar radiation are based on in-site and satellite measurements, the problem of lack of meteorological stations in most places and data privacy in most industrial projects is one of the most important challenges of using RESs. Providing a reliable forecasting model of solar radiation that can also protect data privacy can play a key role in allowing high PV penetration without compromising network performance. In this paper, after reviewing the various aspects of solar radiation forecasting and the methods presented in various studies, a global model of solar radiation forecasting with data privacy was presented. The proposed method is one of the newest approaches to machine learning called federated learning (FL). Independence of the required data in each region and high ability to protect existing data against cyber-attacks and security issues are the most prominent features of the proposed FL procedure. To achieve a global prediction supermodel, the proposed method was trained based on the data of eight scattered regions of Iran with different geographical and meteorological features. The training and test stage for each client in the federated network was performed based on data from each region by one of the deep learning models named convolutional neural network (CNN). The CNN extracted the behavioral pattern of solar radiation related to each region in each client and produced a global supermodel based on the recognized patterns. In order to test and evaluate the performance of the global supermodel produced, which contains the behavioral pattern of solar radiation in various conditions, data related to three new regions of Iran named Abadeh, Jarqavieh, and Arak were used as input dataset for the supermodel. These three regions had no role in the training and generation of the supermodel and were fully used to test the supermodel and express its effectiveness. The presented results were analyzed with various performance evaluation indicators. The result showed that the developed forecasting model was able to forecast solar radiation with a correlation coefficient of 95%, 92%, and 90% for regions Abadeh, Jarqavieh, and Arak, respectively. Finally, in order to evaluate the performance of the developed FL model, four conventional models such as decision tree (DT), back-propagation neural network (BPNN), long short-term memory (LSTM), and bidirectional LSTM (Bi-LSTM) were employed to forecast solar radiation in each of the study regions. It was detected that since no training data were accessible from regions of Abadeh, Jarqavieh, and Arak, the conventional DT, BPNN, LSTM, and Bi-LSTM methods cannot forecast solar radiation in these regions. However, the proposed FL method under the same conditions can present an acceptable solar radiation forecasting of these regions. It should be noted that the developed FL model can be employed as a robust and powerful tool for solving forecasting problems in industrial projects and power or energy systems where data protection is of utmost importance.

## Data Availability

All of the used data in this manuscript are available by request from the corresponding author.
